# Supplementary material for: Thermodynamics-inspired high-entropy oxide synthesis
Source: Nat Commun. 2025 Sep 2;16:8211. doi: 10.1038/s41467-025-63567-z (PMC12405472; doi:10.1038/s41467-025-63567-z)
Supplement: Supplementary file 1 — Supplementary Information [file 41467_2025_63567_MOESM1_ESM.pdf]

Supplementary Information

Thermodynamics-Inspired High-Entropy Oxide Synthesis

Saeed S. I. Almishal[1], Matthew Furst[1], Yueze Tan[1], Jacob T. Sivak[2], Gerald Bejger[3], Joseph Petruska[1], Sai Venkata Gayathri Ayyagari[1], Dhiya Srikanth[1], Nasim Alem[1], Christina M. Rost[3], Susan B. Sinnott[1,2,4], Long-Qing Chen[1], and Jon-Paul Maria[1]

[1]Department of Materials Science and Engineering, The Pennsylvania State University, University Park, PA 16802, USA

[2]Department of Chemistry, The Pennsylvania State University, University Park, PA 16802, USA

[3]Department of Materials Science and Engineering, Virginia Polytechnic Institute and State University, Blacksburg, VA 24061, USA

[4]Institute for Computational and Data Sciences, The Pennsylvania State University, University Park, PA 16802, USA

Corresponding Authors: Saeed S. I. Almishal saeedsialmishal@gmail.com

Keywords: high-entropy oxides, bulk synthesis, equilibrium, binary phase diagrams, oxygen chemical potential, preferred valence, divalent Mn, divalent Fe

Abstract

High-entropy oxide (HEO) thermodynamics transcend temperature-centric approaches, spanning a multidimensional landscape where oxygen chemical potential plays a decisive role. Here, we experimentally demonstrate how controlling the oxygen chemical potential coerces multivalent cations into divalent states in rock salt HEOs. We construct a preferred valence phase diagram based on thermodynamic stability and equilibrium analysis, alongside a high throughput enthalpic stability map derived from atomistic calculations leveraging machine learning interatomic potentials. We identify and synthesize seven equimolar, single-phase rock salt compositions incorporating Mn, Fe, or both, as confirmed by X-ray diffraction and fluorescence. Energy-dispersive X-ray spectroscopy confirms homogeneous cation distribution, while X-ray absorption fine structure analysis reveals predominantly divalent Mn and Fe states, despite their inherent multivalent tendencies. Ultimately, we introduce oxygen chemical potential overlap as a key complementary descriptor predicting HEO stability and synthesizability. Although we focus on rock salt HEOs, our methods are chemically and structurally agnostic, providing a broadly adaptable framework for navigating HEOs thermodynamics and enabling a broader compositional range with contemporary property interest.

Note 1: $Mn_xFe_yO_\delta$ sintered in air compared to their starting precursors

In Figure S1(a), we show the X-ray diffraction (XRD) patterns of the MnO and FeO starting precursors, purchased from Sigma-Aldrich as indicated in the Methods section. Both materials exhibit the expected rock salt structure with no detectable secondary phases. To minimize oxygen and moisture exposure, we vacuum seal the precursor bottles in vacuum bags when not in use and store on a shelf cabinet. Within the resolution of our measurements, we did not observe any changes in the phase purity of the powders over time. In Figure S1(b), we show that sintering MnO, FeO, or their mixtures at 1100°C in air for 5 hours leads to the expected oxidation to higher valence states. FeO oxidizes to Fe_2O_3 adopting the corundum structure, while MnO transforms to Mn_3O_4, forming a tetragonal spinel phase. In mixed MnO-FeO samples, additional peaks appear, indicative of bixbyite and cubic spinel structures.

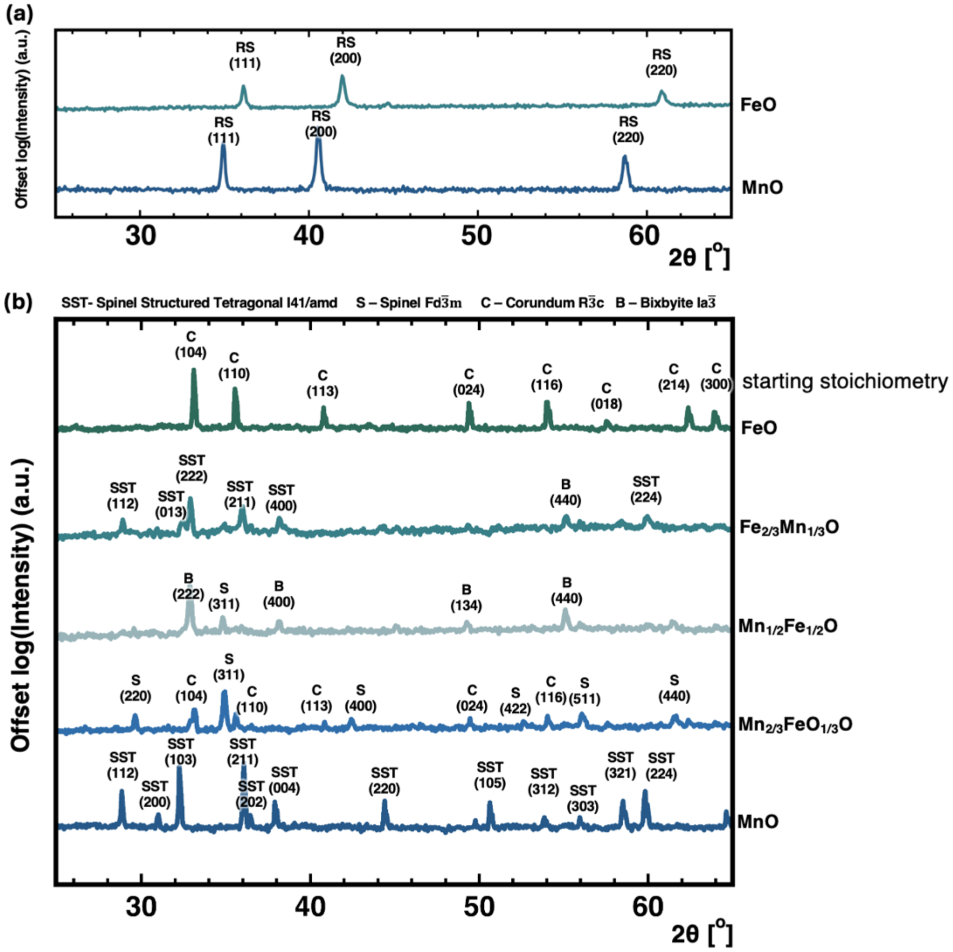

Figure S1. (a) X-ray diffraction (XRD) patterns of MnO and FeO as-received starting precursors, and (b) XRD of MnO-FeO mixtures after sintering at 1100°C in air for 5 hours. Peaks are indexed to their respective crystalline phases.

Note 2: Wide 2θ-θ scans and XRF results for rock salt high entropy oxide compositions

Figure S2 presents the XRD patterns of all single-phase rock salt compositions stabilized by carefully controlling the oxygen partial pressure. For reference and completeness, the prototypical MgCoNiCuZnO composition sintered in air is also included. Note that if MgCoNiCuZnO is sintered, however, at 1100ºC under Ar, CuO reduces as discussed in the main manuscript. Table S1 summarizes the X-ray fluorescence (XRF) fitting results, indicating that the compositions are closely equimolar. Fittings were performed using the Panalytical Omnian platform and reference library, supplemented by eight custom calibrations developed from accurately weighed unreacted powder standards (±0.01 mg accuracy), corresponding to the compositions studied in this work. In our XRF calculations, Mg is fixed at 20% in five-component compositions and 16.667% in the six-component composition, while the remaining cation concentrations are determined from the spectra shown in Figure 3 (main manuscript). We fix the Mg concentration because XRF measurements taken in air cannot reliably quantify its content.

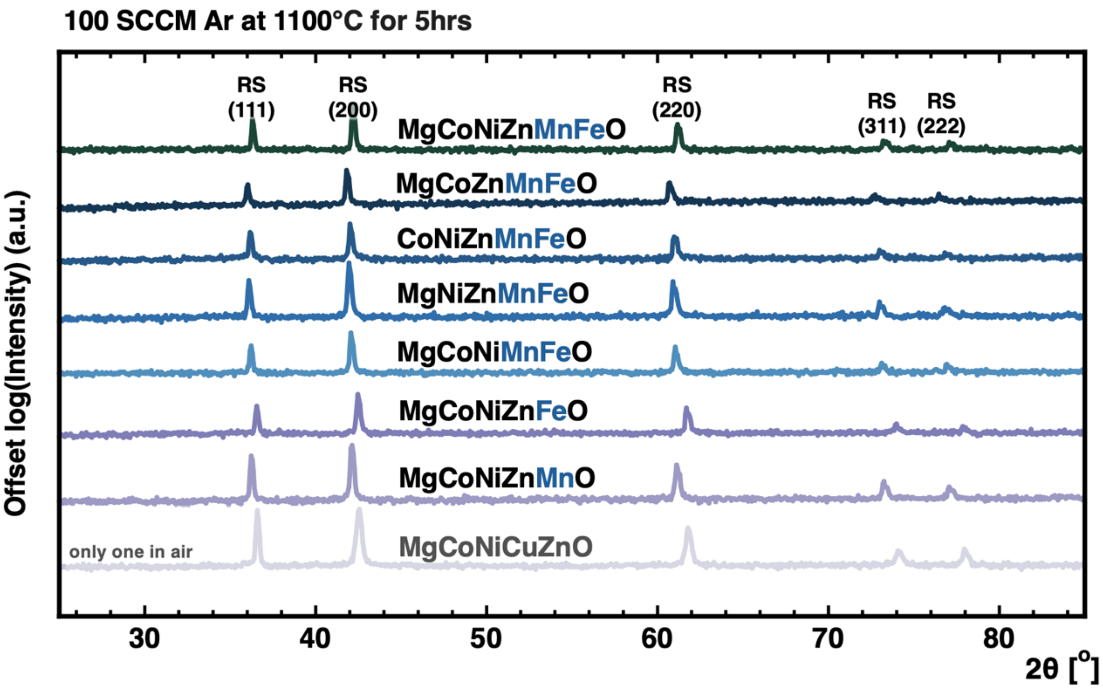

Figure S2. X-ray diffraction patterns of HEO compositions containing Mn, Fe, or both, sintered at 1100°C under 100 SCCM of flowing Ar for 5 hours. All compositions exhibit predominantly rock salt structure characteristic peaks. The prototypical MgCoNiCuZnO sintered in air (ambient pO_2) is added as a reference.

Table S1. Percentage cation concentrations measured from XRF spectra.

Composition	Mg	Co	Ni	Cu	Zn	Mn	Fe
MgCoNiZnMnFeO	16.67	17.14	17.56	0.00	16.35	15.87	16.42
MgCoZnMnFeO	20.00	20.72	0.00	0.00	18.21	20.24	20.83
CoNiZnMnFeO	0.00	21.24	20.90	0.00	18.40	21.14	18.33
MgNiZnMnFeO	20.00	0.00	21.41	0.00	16.00	21.19	21.40
MgCoNiMnFeO	20.00	19.60	19.69	0.00	0.00	18.61	22.11
MgCoNiZnMnO	20.00	20.53	21.07	0.00	16.84	21.56	0.00
MgCoNiCuZnO	20.00	20.96	19.56	18.21	21.27	0.00	0.00

Note 3: X-ray absorption near edge structure (XANES) and their derivatives

Figures S3-S5 along with Figure 4 and Figure 5(e) in the manuscript present the full set of collected XANES spectra alongside reference standards, providing the basis for accurate determination of E_0, all E_0 values are summarized in Table S2. The corresponding derivative spectra are also included to aid in identifying the transition energy. Figure S3 illustrates Co and Ni K-edge data, while Figure S4 shows only the derivative curves for the Mn and Fe K-edges presented in Figure 4 for the 5-component MgNiZnMnFeO and CoNiZnMnFeO. Figure S5 illustrates the complete spectra set for the parent composition MgCoNiZnMnFeO. To ensure consistent tracking of binding energies and reliable valence-state assignments, we define E_0 based on the same electronic transition identified from the derivative spectra across all samples, with the oxidation state increasing as the excitation energy required for the transition increases.

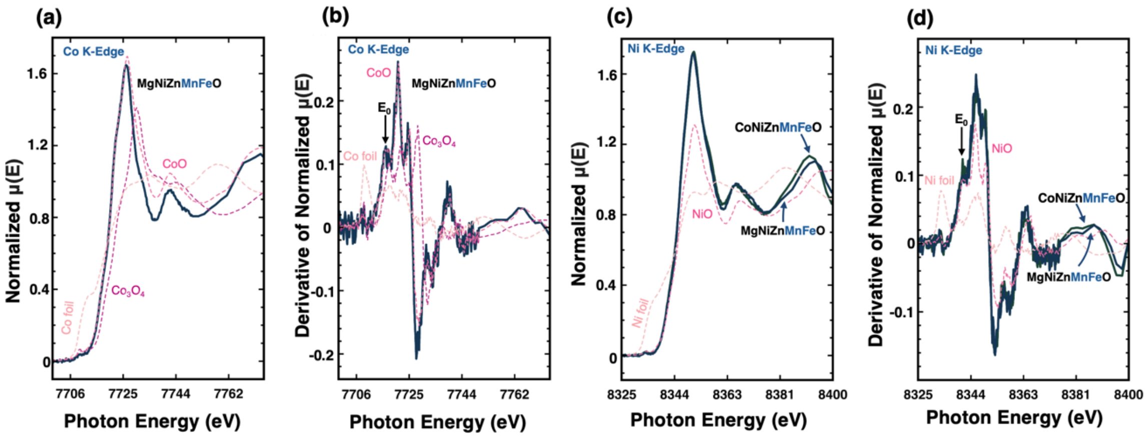

Figure S3. (a) XANES spectra of the Co K-edge for MgNiZnMnFeO and CoNiZnMnFeO, compared to reference spectra, with their derivatives shown in (b). (c) XANES spectra at of the Ni K-edge for MgNiZnMnFeO and CoNiZnMnFeO, compared to reference spectra, with the corresponding spectral derivative in (d).

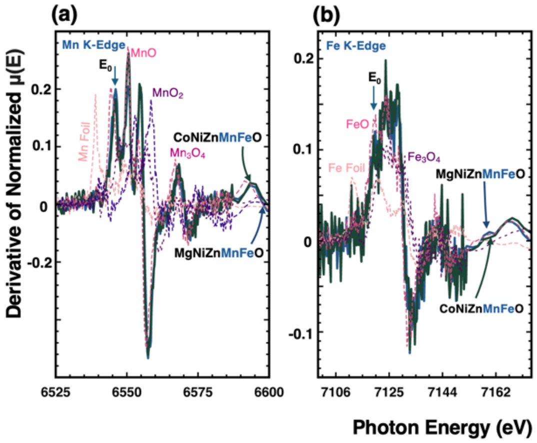

Figure S4. (a) First derivative of XANES spectra at the Mn K-edge for MgNiZnMnFeO and CoNiZnMnFeO, compared to reference spectra, corresponding to Figure 4(a), with MnO_2 included as an additional reference. (b) First derivative of the XANES spectra at the Fe K-edge for MgNiZnMnFeO and CoNiZnMnFeO, compared to reference spectra, corresponding to Figure 4(c).

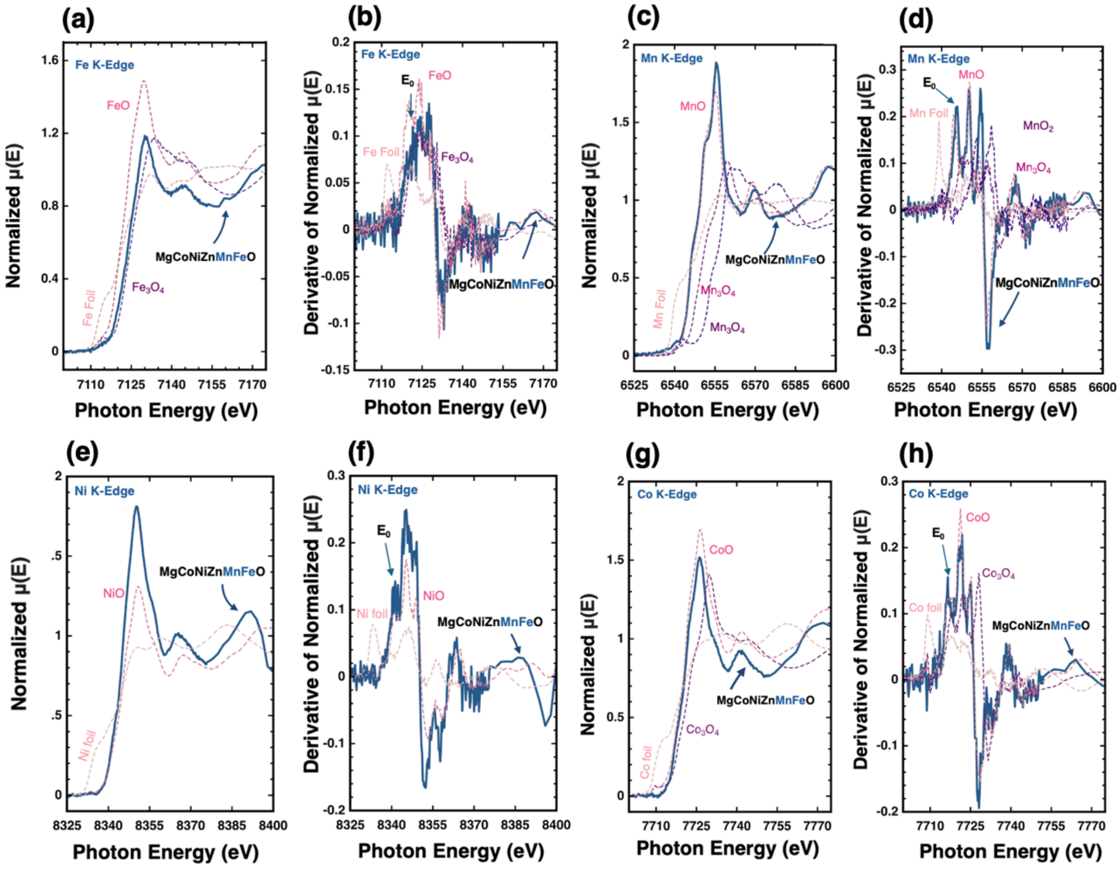

Figure S5. XANES spectra for the parent composition MgCoNiZnMnFeO, compared to reference standards: (a) Fe K-edge, (c) Mn K-edge, (e) Ni K-edge, and (g) Co K-edge. Corresponding first derivatives are shown in (b), (d), (f), and (h), respectively, for accurate determination of edge positions. The extracted Mn and Fe valence states, based on these data, are illustrated in Figure 5(e) of the main manuscript.

Table S2. Summary of measured edge energy values (in eV) and their corresponding valence states

Composition	Valence state	Measured Edge energy value (eV)
**Mn K-edge**		
Mn	0+	6538.96
MnO	2+	6544.42
Mn_3O_4	2.667+	6547.45
MnO_2	4+	6552.94
MgZnNiMnFeO	~2.2+ (from best linear fit)	6546.05
CoNiZnMnFeO	~2.2+ (from best linear fit)	6546.06
MgCoNiZnMnFeO	~2.065 (from best linear fit)	6545.59
**Fe K-edge**		
Fe	0+	7111.94
FeO	2+	7119.67
Fe_3O_4	2.667+	7122.91
MgZnNiMnFeO	~2.03+ (from best linear fit)	7120.11
CoNiZnMnFeO	~2.18+ (from best linear fit)	7121.18
MgCoNiZnMnFeO	~2.087 (from best linear fit)	7120.33

Note 4: More stringent reducing conditions: MgCoNiMnFeO as an additional example

In Figure 5(b) in the main manuscript, we show that more stringent reducing condition by adding 1% H_2 to the Ar mixture results in reducing some cations and forming a metallic phase in MgCoNiZnMnFeO. Figure S6 provides further support of this behavior in the five-component composition MgCoNiMnFeO, and similar behavior is expected across all studied compositions.

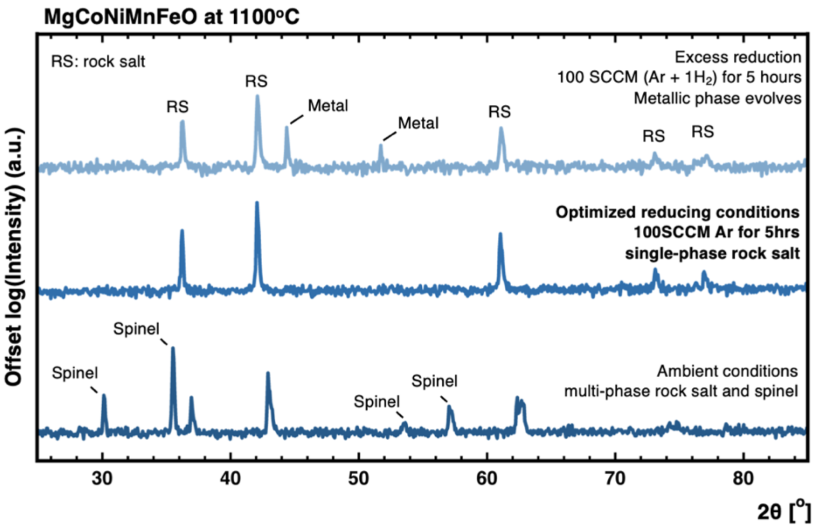

Figure S6. X-ray diffraction patterns $Mg_{1/5}Co_{1/5}Ni_{1/5}Mn_{1/5}Fe_{1/5}O$ sintered at 1100°C under different oxygen partial pressure. Synthesis under forming gas (Ar +1%H_2) result in reduced FCC metallic phase, most probably associated with Ni and Co reducing.

Note 5: Additional EDX spectra for MgCoNiCuZnMnFeO at 100nm and 500nm scale

In addition to the 50 nm scale EDS maps for MgCoNiCuZnMnFeO presented in the main manuscript (Figure 5(d)), Figure S7 summarizes complementary EDS maps acquired at 100 nm and 500 nm scales. These results demonstrate that the cations remain homogeneously distributed even at those larger length scales, with no detectable signatures of clustering or segregation.

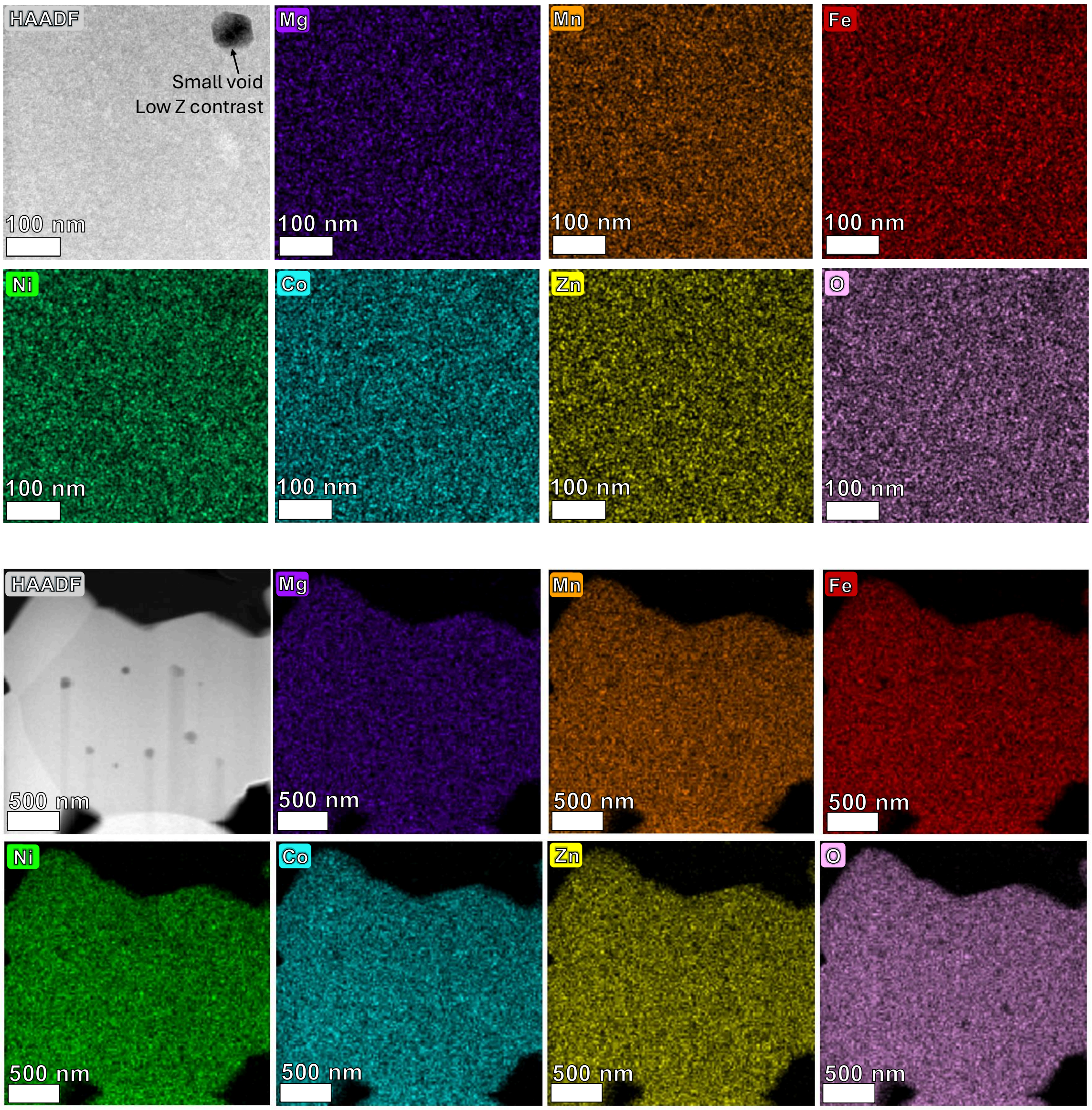

Figure S7. Energy-dispersive spectroscopy (EDS) maps showing a homogeneous distribution of cations in MgCoNiZnMnFeO at the 100nm (top panels) and 500nm (bottom panels) scale.

Note 6: Chemical potential diagrams

Figure S6 presents the chemical potentials of 3d transition metals and Mg, extracted from The Materials Project database. The green-bolded region highlights the chemical potential range where $A^{2+}O^{2-}$ is thermodynamically stable. Notably, Sc, V, and Cr do not exhibit a stable $A^{2+}O^{2-}$ phase on the convex hull at 0K, whereas Ti forms a stable phase only at very low oxygen chemical potential.

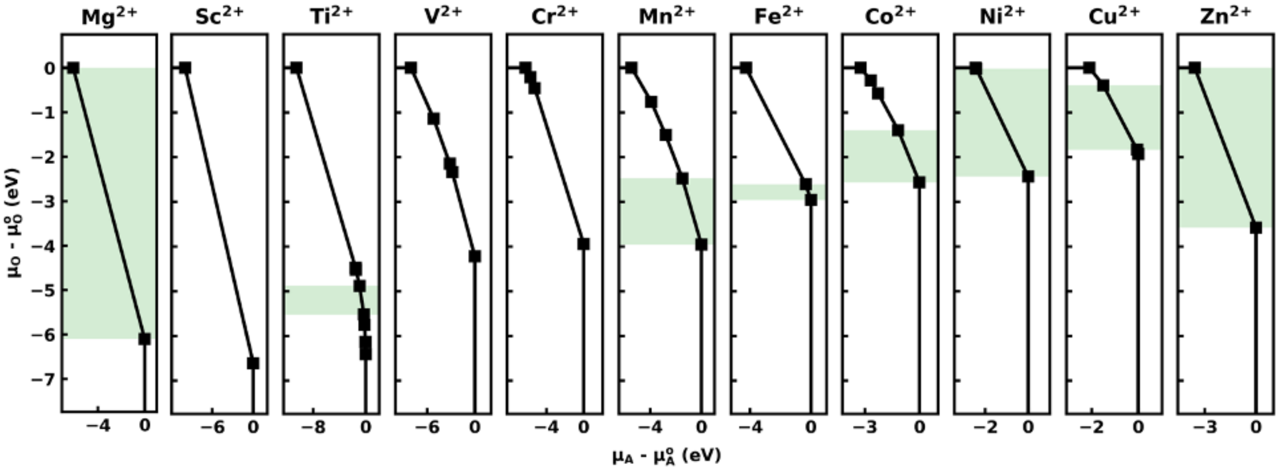

Figure S8. A-O chemical potential diagrams for Mg and all 3d transition metals explored in this study. A green shade denotes oxygen chemical potential regions in which $A^{2+}O^{2-}$ compositions are stable.

Note 7: Chemical potential overlap descriptor

In Table S2, we summarize the values for the computational descriptors for equimolar HEO compositions from the cation cohort: Mg, Mn, Fe, Co, Ni, Cu, and Zn. Compositions are sorted by the oxygen chemical potential overlap descriptor proposed in this work, $\mu_{overlap}$. The refined single-phase stability thresholds are defined from our previous work[1] to determine predicted single-phase stability thresholds (ΔH_{mix} = 92.2 meV/atom, σ_{bonds} = 0.102 Å) as well as $\mu_{overlap}$ = -0.191 eV. Notably, a positive value for $\mu_{overlap}$ indicates an overlap in oxygen chemical potential space (a wider synthesis window), while a negative value indicates separation (a narrow/impossible synthesis window). Predicted and experimental stability are indicated as single- and multi-phase with circles and crosses, respectively. Compositions not yet explored experimentally are indicated by two dashes. A complete set of values, including those for Ca, is provided in our previous work (see Ref.[1]).

Accurately determining the corresponding experimental synthesis atmosphere from $\mu_{overlap}$, however, requires extending the descriptor to incorporate temperature and experimentally verified oxygen partial pressure reference values. Nonetheless, comparing chemical potential overlaps under different conditions allows qualitative prediction of whether reducing, inert, or oxidizing environments are needed. For example, in Figure S8, MgCoNiCuZnO exhibits an overlap between -1.4 and -1.84 eV along the oxygen chemical potential axis, while MnO and FeO require potentials

below -2.48 eV to stabilize. Since, generally for ideal gases, chemical potential is proportional to the logarithm of partial pressure, lower chemical potentials in this case correspond to lower oxygen partial pressures, suggesting that more reducing conditions are needed to stabilize Mn- and Fe-containing compositions compared to MgCoNiCuZnO.

Additionally, we can quantitatively estimate the synthesis conditions using the ideal gas relationship: $\ln\left(\frac{P}{P_{ref}}\right) = \frac{2\Delta\mu_O}{RT}$,where P_{ref} is the oxygen partial pressure under ambient conditions (0.21 atm for MgCoNiCuZnO), and $\Delta\mu_O$ is the minimum difference in chemical potential between the composition of interest and the reference composition. For example, the first appearance of Mn^{2+} occurs at -2.48 eV and that of Fe^{2+} at -2.61 eV in Figure S8, resulting in $\Delta\mu_O$ values of -0.64 eV and -0.77 eV, relative to MgCoNiCuZnO respectively. Solving for the required oxygen partial pressure at 1100°C (1373 K) yields target values of 4.18×10^{-6} atm for Mn^{2+} stabilization and 4.69×10^{-7} atm for Fe^{2+} stabilization. These values represent the maximum allowable oxygen partial pressures for phase stabilization, suggesting that actual synthesis should proceed under more reducing conditions. This approximation aligns with our binary phase diagram predictions and synthesis approach.

Table S3. Computational descriptors for equimolar HEO compositions. ΔH_{mix} and σ_{bonds} values are obtained with permission from ref [1]. Crosses denote unstable compositions, circles indicate stable compositions, and dashes represent compositions that, to the best of our knowledge, have not been reported at the time of publication.

**Composition**	**$\mu_{overlap}$ (eV)**	**ΔH_{mix} (eV/atom)**	**σ_{bonds} (Å)**	**Pred. Stability**	**Expt. Stability**
$Mg_{1/4}Ni_{1/4}Cu_{1/4}Zn_{1/4}O$	1.443	0.097	0.102	✖	✖ [2]
$Mg_{1/4}Co_{1/4}Ni_{1/4}Zn_{1/4}O$	1.035	0.079	0.020	●	● [2]
$Mg_{1/4}Co_{1/4}Ni_{1/4}Cu_{1/4}O$	0.436	0.077	0.099	●	● [2]
$Mg_{1/4}Co_{1/4}Cu_{1/4}Zn_{1/4}O$	0.436	0.093	0.116	✖	✖ [2]
$Co_{1/4}Ni_{1/4}Cu_{1/4}Zn_{1/4}O$	0.436	0.100	0.116	✖	✖ [2]
$Mg_{1/5}Co_{1/5}Ni_{1/5}Cu_{1/5}Zn_{1/5}O$	0.436	0.091	0.087	●	● [3]
$Mg_{1/4}Mn_{1/4}Fe_{1/4}Zn_{1/4}O$	0.336	0.070	0.042	●	--
$Mg_{1/4}Mn_{1/4}Co_{1/4}Zn_{1/4}O$	0.091	0.072	0.037	●	--
$Mg_{1/4}Mn_{1/4}Co_{1/4}Ni_{1/4}O$	-0.041	0.060	0.036	●	--
$Mg_{1/4}Mn_{1/4}Ni_{1/4}Zn_{1/4}O$	-0.041	0.061	0.039	●	--
$Mn_{1/4}Co_{1/4}Ni_{1/4}Zn_{1/4}O$	-0.041	0.079	0.036	●	--
$Mg_{1/5}Mn_{1/5}Co_{1/5}Ni_{1/5}Zn_{1/5}O$	-0.041	0.071	0.036	●	● [this work]
$Mg_{1/4}Mn_{1/4}Fe_{1/4}Co_{1/4}O$	-0.059	0.057	0.036	●	--
$Mg_{1/4}Fe_{1/4}Co_{1/4}Zn_{1/4}O$	-0.059	0.075	0.024	●	--
$Mn_{1/4}Fe_{1/4}Co_{1/4}Zn_{1/4}O$	-0.059	0.078	0.030	●	--

$Mg_{1/5}Mn_{1/5}Fe_{1/5}Co_{1/5}Zn_{1/5}O$	-0.059	0.071	0.035	●	● [this work]
$Mg_{1/4}Mn_{1/4}Fe_{1/4}Ni_{1/4}O$	-0.191	0.053	0.042	●	--
$Mg_{1/4}Fe_{1/4}Co_{1/4}Ni_{1/4}O$	-0.191	0.071	0.026	●	--
$Mg_{1/4}Fe_{1/4}Ni_{1/4}Zn_{1/4}O$	-0.191	0.068	0.027	●	--
$Mn_{1/4}Fe_{1/4}Co_{1/4}Ni_{1/4}O$	-0.191	0.059	0.036	●	--
$Mn_{1/4}Fe_{1/4}Ni_{1/4}Zn_{1/4}O$	-0.191	0.076	0.039	●	--
$Fe_{1/4}Co_{1/4}Ni_{1/4}Zn_{1/4}O$	-0.191	0.090	0.025	●	--
$Mg_{1/5}Mn_{1/5}Fe_{1/5}Co_{1/5}Ni_{1/5}O$	-0.191	0.062	0.036	●	● [4] [this work]
$Mg_{1/5}Mn_{1/5}Fe_{1/5}Ni_{1/5}Zn_{1/5}O$	-0.191	0.066	0.039	●	● [this work]
$Mg_{1/5}Fe_{1/5}Co_{1/5}Ni_{1/5}Zn_{1/5}O$	-0.191	0.077	0.025	●	● [this work]
$Mn_{1/5}Fe_{1/5}Co_{1/5}Ni_{1/5}Zn_{1/5}O$	-0.191	0.076	0.035	●	● [this work]
$Mg_{1/6}Mn_{1/6}Fe_{1/6}Co_{1/6}Ni_{1/6}Zn_{1/6}O$	-0.191	0.071	0.036	●	● [this work]
$Mg_{1/4}Mn_{1/4}Co_{1/4}Cu_{1/4}O$	-0.640	0.057	0.128	✖	--
$Mg_{1/4}Mn_{1/4}Ni_{1/4}Cu_{1/4}O$	-0.640	0.053	0.115	✖	--
$Mg_{1/4}Mn_{1/4}Cu_{1/4}Zn_{1/4}O$	-0.640	0.075	0.137	✖	--
$Mn_{1/4}Co_{1/4}Ni_{1/4}Cu_{1/4}O$	-0.640	0.050	0.126	✖	--
$Mn_{1/4}Co_{1/4}Cu_{1/4}Zn_{1/4}O$	-0.640	0.080	0.149	✖	--
$Mn_{1/4}Ni_{1/4}Cu_{1/4}Zn_{1/4}O$	-0.640	0.079	0.136	✖	--
$Mg_{1/5}Mn_{1/5}Co_{1/5}Ni_{1/5}Cu_{1/5}O$	-0.640	0.060	0.100	✖	--
$Mg_{1/5}Mn_{1/5}Co_{1/5}Cu_{1/5}Zn_{1/5}O$	-0.640	0.075	0.114	✖	--
$Mg_{1/5}Mn_{1/5}Ni_{1/5}Cu_{1/5}Zn_{1/5}O$	-0.640	0.073	0.104	✖	--
$Mn_{1/5}Co_{1/5}Ni_{1/5}Cu_{1/5}Zn_{1/5}O$	-0.640	0.076	0.114	✖	--
$Mg_{1/6}Mn_{1/6}Co_{1/6}Ni_{1/6}Cu_{1/6}Zn_{1/6}O$	-0.640	0.074	0.091	✖	--
$Mg_{1/4}Mn_{1/4}Fe_{1/4}Cu_{1/4}O$	-0.790	0.048	0.135	✖	--
$Mg_{1/4}Fe_{1/4}Co_{1/4}Cu_{1/4}O$	-0.790	0.063	0.119	✖	--
$Mg_{1/4}Fe_{1/4}Ni_{1/4}Cu_{1/4}O$	-0.790	0.060	0.107	✖	--
$Mg_{1/4}Fe_{1/4}Cu_{1/4}Zn_{1/4}O$	-0.790	0.073	0.127	✖	--
$Mn_{1/4}Fe_{1/4}Co_{1/4}Cu_{1/4}O$	-0.790	0.047	0.139	✖	--
$Mn_{1/4}Fe_{1/4}Ni_{1/4}Cu_{1/4}O$	-0.790	0.045	0.135	✖	--
$Mn_{1/4}Fe_{1/4}Cu_{1/4}Zn_{1/4}O$	-0.790	0.073	0.157	✖	--
$Fe_{1/4}Co_{1/4}Ni_{1/4}Cu_{1/4}O$	-0.790	0.062	0.118	✖	--
$Fe_{1/4}Co_{1/4}Cu_{1/4}Zn_{1/4}O$	-0.790	0.087	0.135	✖	--
$Fe_{1/4}Ni_{1/4}Cu_{1/4}Zn_{1/4}O$	-0.790	0.087	0.125	✖	--
$Mg_{1/5}Mn_{1/5}Fe_{1/5}Co_{1/5}Cu_{1/5}O$	-0.790	0.055	0.114	✖	--
$Mg_{1/5}Mn_{1/5}Fe_{1/5}Ni_{1/5}Cu_{1/5}O$	-0.790	0.052	0.110	✖	--

$Mg_{1/5}Mn_{1/5}Fe_{1/5}Cu_{1/5}Zn_{1/5}O$	-0.790	0.067	0.121	✖	--
$Mg_{1/5}Fe_{1/5}Co_{1/5}Ni_{1/5}Cu_{1/5}O$	-0.790	0.067	0.094	✖	--
$Mg_{1/5}Fe_{1/5}Co_{1/5}Cu_{1/5}Zn_{1/5}O$	-0.790	0.077	0.104	✖	--
$Mg_{1/5}Fe_{1/5}Ni_{1/5}Cu_{1/5}Zn_{1/5}O$	-0.790	0.075	0.096	✖	--
$Mn_{1/5}Fe_{1/5}Co_{1/5}Ni_{1/5}Cu_{1/5}O$	-0.790	0.051	0.114	✖	--
$Mn_{1/5}Fe_{1/5}Co_{1/5}Cu_{1/5}Zn_{1/5}O$	-0.790	0.072	0.127	✖	--
$Mn_{1/5}Fe_{1/5}Ni_{1/5}Cu_{1/5}Zn_{1/5}O$	-0.790	0.071	0.121	✖	--
$Fe_{1/5}Co_{1/5}Ni_{1/5}Cu_{1/5}Zn_{1/5}O$	-0.790	0.084	0.104	✖	--
$Mg_{1/6}Mn_{1/6}Fe_{1/6}Co_{1/6}Ni_{1/6}Cu_{1/6}O$	-0.790	0.058	0.094	✖	--
$Mg_{1/6}Mn_{1/6}Fe_{1/6}Co_{1/6}Cu_{1/6}Zn_{1/6}O$	-0.790	0.069	0.104	✖	--
$Mg_{1/6}Mn_{1/6}Fe_{1/6}Ni_{1/6}Cu_{1/6}Zn_{1/6}O$	-0.790	0.067	0.098	✖	--
$Mg_{1/6}Fe_{1/6}Co_{1/6}Ni_{1/6}Cu_{1/6}Zn_{1/6}O$	-0.790	0.078	0.083	✖	--
$Mn_{1/6}Fe_{1/6}Co_{1/6}Ni_{1/6}Cu_{1/6}Zn_{1/6}O$	-0.790	0.071	0.104	✖	--

Note 8: Chemical potential overlap descriptor applied to rare earth sesquioxides and fluorites

In the abstract, we refer to our framework as structurally and chemically agnostic, meaning it can extend beyond 2+ cations and the rock salt structure to more complex chemistries and crystal structures. To demonstrate this broader applicability, we apply the $\mu_{overlap}$ framework to the rare-earth system $(Ce,La,Pr,Sm,Y)_2O_{3+\delta}$; a composition known for its complex oxidation behavior, oxygen nonstoichiometry, and structural polymorphism, as explored in the influential studies by Djenadic et al.[5]and Sarkar et al.[6], and more recently by Kotsonis et al.[7]. We further extend the framework to the $(Zr,Ce,La,Pr,Sm,Y)O_{2-\delta}$ composition by incorporating ZrO_2.

Figure S9(a) shows the oxygen chemical potential diagrams, similar to those in Figure S8, for Y-, La-, Pr-, and Sm-oxides, highlighting the stability windows of their $(A^{3+})_2O_3$ binaries, as well as ZrO_2, reflecting its unwavering 4+ stability over a ~5 eV window. Ce–O, however, is treated separately to capture its accessible oxidation states from Ce^{3+} to Ce^{4+}. Figure S9(b) depicts the corresponding $\mu_{overlap}$ descriptor heat map. Starting with the five-component, $(Ce,La,Pr,Sm,Y)_2O_{3+\delta}$ composition, the corresponding $\mu_{overlap}$ map (Figure S9b) reveals that both Ce^{3+} and Ce^{4+} exhibit overlap with the trivalent rare-earth cations; however, Ce^{4+} displays a larger $\mu_{overlap}$ and is thermodynamically favored at high oxygen chemical potentials. Our simple model therefore predicts a majority Ce^{4+} character, while the remaining cations retain their trivalent states under ambient pO_2 conditions consistent with experimentally reported oxidation states for this system[8].

Oxidation state predictions based on $\mu_{overlap}$ provide further insight: assuming full Ce^{4+} occupancy, the average cation oxidation state in $(Ce,La,Pr,Sm,Y)_2O_{3+\delta}$ is 3.2, corresponding to a

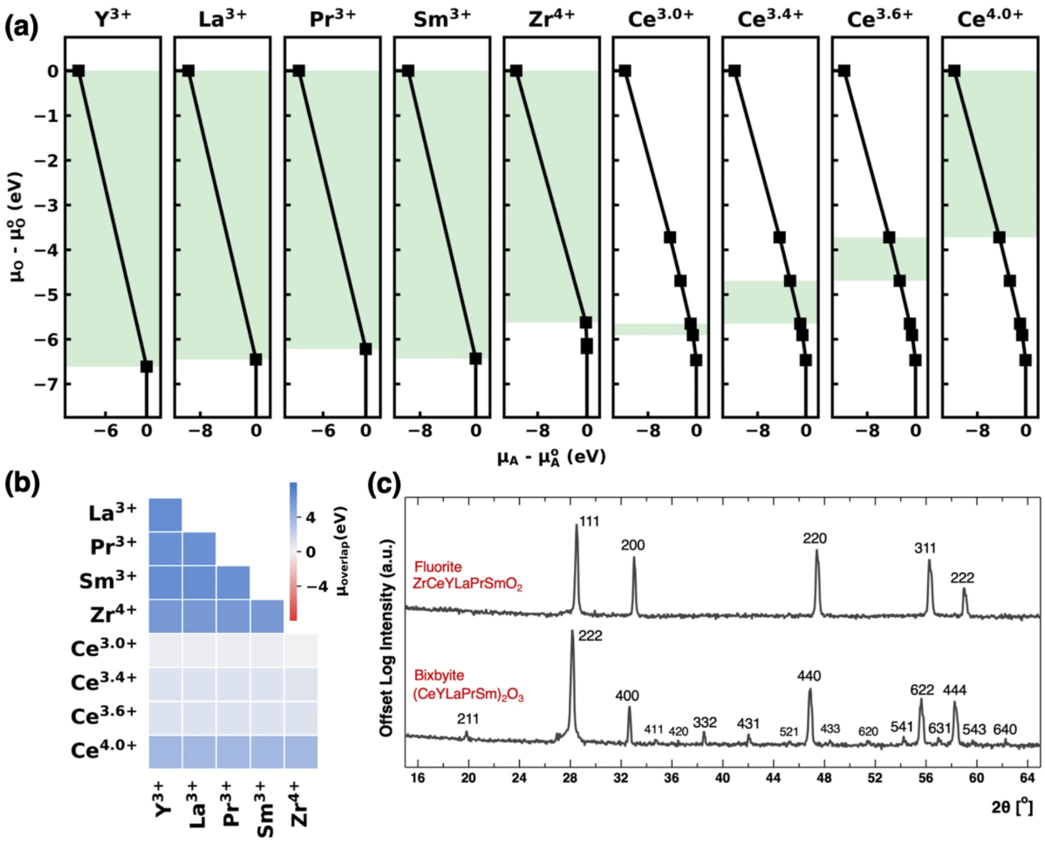

Figure S9. (a) Oxygen chemical potential diagrams for Y-, La-, Pr-, and Sm-oxides, highlighting the stability windows of their $(A^{3+})_2O_3$ binaries, and for ZrO_2, reflecting its 4+ stability. Ce–O is shown separately to capture its multiple accessible oxidation states. Green shaded regions indicate oxygen chemical potential ranges where the desired stoichiometries are thermodynamically stable. All data are extracted from the Materials Project database. (b) $\mu_{overlap}$ heat map for all two-cation combinations, illustrating the range of overlap values for Ce depending on its oxidation state, and the strong compatibility between Zr^{4+} and both Ce^{4+} and the trivalent cations. (c) X-ray diffraction (XRD) patterns of $(Ce,La,Pr,Sm,Y)_2O_3$ (bixbyite) and $Zr(Ce,La,Pr,Sm,Y)O_2$ (defective fluorite) compositions. Samples were sintered at 1400 °C after 18 hours of wet milling with 3 mm YSZ balls and methanol.

nominal $(Ce,La,Pr,Sm,Y)_2O_{3.2}$ composition. Although this stoichiometry does not exactly match bixbyite (A_2O_3), it more closely approaches the oxygen content and cation-to-oxygen ratio characteristic of bixbyite than fluorite. While enthalpy-stabilized bixbyite typically requires an ordered oxygen sublattice and resists accommodating Ce^{4+}, high-entropy configurations enable local Ce^{4+} deviations, while the surrounding trivalent cations (Y^{3+}, La^{3+}, Pr^{3+}, Sm^{3+}) maintain an overall 3+ network, preserving bixbyite symmetry, as confirmed by X-ray diffraction (Figure S9(c)) and XANES analysis[8]. Even if the argument were reversed and we began with bixbyite symmetry revealed by XRD, the $\mu_{overlap}$ framework, by virtue of its structure-agnostic nature, would still insist on majority Ce^{4+} character, exposing valence complexities not apparent from structure descriptors alone.

We next consider introducing Zr^{4+} into the composition. Zr^{4+} increases the chemical potential overlap (Figure S9b), further supporting Ce^{4+} and raising the average cation valence to

~3.33+, shifting the nominal stoichiometry toward $A_2O_{3.33}$ or more accurately $AO_{1.667}$, characteristic of a defective fluorite structure. X-ray diffraction (Figure S9c) confirms this structural transition, showing that Zr addition drives the system from a bixbyite to a defective fluorite phase, consistent with predictions from the $\mu_{overlap}$ framework. While $\mu_{overlap}$ does not directly predict crystal structure, it complements thermodynamic descriptors, such as ΔH_{mix} and σ_{bonds}, which are essential for capturing this structural transformation. Nonetheless, $\mu_{overlap}$ narrows accessible phase space and informs stability and synthesizability by predicting the average stable cation oxidation states.

Although the current set of descriptors offers strong predictive capabilities, synthesis is ultimately constrained by available materials, laboratory-accessible boundary conditions, and kinetic factors, which can drive systems away from their predicted thermodynamic equilibrium states. For instance, Pr is often commercially available as Pr_6O_{11}, containing significant Pr^{4+}, which can shift the oxidation balance and influence phase stability. Additionally, although $(Ce,La,Pr,Sm,Y)_2O_3$ adopts the bixbyite structure under equilibrium conditions, nonequilibrium pathways often favor defective fluorite formation[7], reflecting the competition between ordered oxygen lattice sites stabilization and configurational entropy gains from disordered unoccupied oxygen lattice sites[7]. Nevertheless, these predictive descriptors bring us closer to fully realizing a comprehensive framework for predictive synthesis.

Note 9: Outlook on Kinetic factors, far-from-equilibrium synthesis, and emerging functionality

Although $\mu_{overlap}$ is grounded in equilibrium thermodynamics, it provides valuable guidance even for far-from-equilibrium synthesis methods such as pulsed laser deposition, magnetron sputtering, and joule heating, which access alternative kinetic pathways and stabilize valence states and structures not achievable under equilibrium conditions. For instance, in our previous work on MgCoNiCuZnO thin films grown by pulsed laser deposition[9,10], we observed Co^{3+} formation, consistent with $\mu_{overlap}$ and chemical potential diagrams: Co^{3+} stabilizes at higher oxygen chemical potentials, overlapping with the divalent cations' stability window, where Co_3O_4 (nominally $Co^{2.67}$, with a 1:2 ratio of Co^{2+} to Co^{3+}) lies just above the divalent stability region (green shade) in Figure S8. This behavior is also consistent with binary phase diagrams, where $Co^{2.67}$ becomes favored at higher oxygen pressures or lower synthesis temperatures. To explicitly address kinetic and non-ideal thermodynamic complexities, in our other work we integrate phase-field modeling to simulate ordering and local structure evolution[9,11,12], decomposition enthalpy to predict metastable phase stability[2], and cluster expansion to capture short-range ordering effects[13]. Ongoing efforts focus on extending these models to incorporate defects, valence dynamics, and charge transfer. While $\mu_{overlap}$ does not account for kinetics directly, it complements these approaches, offering a broader predictive framework. Here, we use the chemical potential diagrams and $\mu_{overlap}$ framework

in its simplest forms to demonstrate its broad applicability, but it has much greater potential that will be the focus of future work.

Beyond stabilizing Mn and Fe in the divalent state, their multivalency offers access to a broader valence landscape under non-equilibrium conditions, potentially enabling control over charge trapping, ion migration, and resistive switching[2,13,14]. Their incorporation into the rock salt HEO family is also expected to introduce emergent magnetic interactions[4,15,16], opening pathways for multifunctional HEOs design. The seven compositions synthesized here may thus offer a foundation for future studies exploring magnetism, polaron conduction, and related functionalities in HEOs.

Supplementary References

(1) Sivak, J. T.; Almishal, S. S. I.; Caucci, M. K.; Tan, Y.; Srikanth, D.; Petruska, J.; Furst, M.; Chen, L.-Q.; Rost, C. M.; Maria, J.-P.; Sinnott, S. B. Discovering High-Entropy Oxides with a Machine-Learning Interatomic Potential. *Phys. Rev. Lett.* **2025**, *134* (21), 216101. https://doi.org/10.1103/PhysRevLett.134.216101.

(2) Almishal, S. S. I.; Sivak, J. T.; Kotsonis, G. N.; Tan, Y.; Furst, M.; Srikanth, D.; Crespi, V. H.; Gopalan, V.; Heron, J. T.; Chen, L.-Q.; Rost, C. M.; Sinnott, S. B.; Maria, J.-P. Untangling Individual Cation Roles in Rock Salt High-Entropy Oxides. *Acta Materialia* **2024**, *279*, 120289. https://doi.org/10.1016/j.actamat.2024.120289.

(3) Rost, C. M.; Sachet, E.; Borman, T.; Moballegh, A.; Dickey, E. C.; Hou, D.; Jones, J. L.; Curtarolo, S.; Maria, J.-P. Entropy-Stabilized Oxides. *Nature Communications* **2015**, *6* (1), 8485. https://doi.org/10.1038/ncomms9485.

(4) Pu, Y.; Moseley, D.; He, Z.; Pitike, K. C.; Manley, M. E.; Yan, J.; Cooper, V. R.; Mitchell, V.; Peterson, V. K.; Johannessen, B.; Hermann, R. P.; Cao, P. (Mg,Mn,Fe,Co,Ni)O: A Rocksalt High-Entropy Oxide Containing Divalent Mn and Fe. *Sci. Adv.* **2023**, *9* (38), eadi8809. https://doi.org/10.1126/sciadv.adi8809.

(5) Djenadic, R. Multicomponent Equiatomic Rare Earth Oxides. *Materials Research Letters* **2017**, *5*, 102–109.

(6) Sarkar, A.; Djenadic, R.; Usharani, N. J.; Sanghvi, K. P.; Chakravadhanula, V. S. K.; Gandhi, A. S.; Hahn, H.; Bhattacharya, S. S. Nanocrystalline Multicomponent Entropy Stabilised Transition Metal Oxides. *Journal of the European Ceramic Society* **2017**, *37* (2), 747–754. https://doi.org/10.1016/j.jeurceramsoc.2016.09.018.

(7) Kotsonis, G. N.; Almishal, S. S. I.; Miao, L.; Caucci, M. K.; Bejger, G. R.; Ayyagari, S. V. G.; Valentine, T. W.; Yang, B. E.; Sinnott, S. B.; Rost, C. M.; Alem, N.; Maria, J.-P. Fluorite-Structured High-Entropy Oxide Sputtered Thin Films from Bixbyite Target. *Applied Physics Letters* **2024**, *124* (17), 171901. https://doi.org/10.1063/5.0201419.

(8) Bejger, G. R.; Caucci, M. K.; Almishal, S. S. I.; Yang, B.; Maria, J.-P.; Sinnott, S. B.; Rost, C. M. *Lanthanide L-Edge Spectroscopy of High-Entropy Oxides: Insights into Valence and Phase Stability*. arXiv.org. https://arxiv.org/abs/2505.08055v1 (accessed 2025-06-07).

(9) Almishal, S. S. I.; Miao, L.; Tan, Y.; Kotsonis, G. N.; Sivak, J. T.; Alem, N.; Chen, L.-Q.; Crespi, V. H.; Dabo, I.; Rost, C. M.; Sinnott, S. B.; Maria, J.-P. Order Evolution from a High-Entropy Matrix: Understanding and Predicting Paths to Low-Temperature Equilibrium. *Journal of the American Ceramic Society* e20223. https://doi.org/10.1111/jace.20223.

(10) Kotsonis, G. N.; Meisenheimer, P. B.; Miao, L.; Roth, J.; Wang, B.; Shafer, P.; Engel-Herbert, R.; Alem, N.; Heron, J. T.; Rost, C. M.; Maria, J.-P. Property and Cation Valence Engineering in

Entropy-Stabilized Oxide Thin Films. *Phys. Rev. Mater.* **2020**, *4* (10), 100401. https://doi.org/10.1103/PhysRevMaterials.4.100401.

(11) Tan, Y.; Sivak, J. T.; Almishal, S. S. I.; Maria, J.-P.; Sinnott, S. B.; Ji, Y.; Chen, L.-Q. Phase-Field Study of Precipitate Morphology in Epitaxial High-Entropy Oxide Films. *Acta Materialia* **2025**, *286*, 120721. https://doi.org/10.1016/j.actamat.2025.120721.

(12) Niculescu, G. E.; Bejger, G. R.; Barber, J. P.; Wright, J. T.; Almishal, S. S. I.; Webb, M.; Ayyagari, S. V. G.; Maria, J.-P.; Alem, N.; Heron, J. T.; Rost, C. M. Local Structure Maturation in High Entropy Oxide (Mg,Co,Ni,Cu,Zn)1-x(Cr,Mn)xO Thin Films. arXiv June 19, 2024. https://doi.org/10.48550/arXiv.2406.13550.

(13) Almishal, S. S. I.; Kezer, P.; Sivak, J. T.; Iwabuchi, Y.; Ayyagari, S. V. G.; Sarker, S.; Furst, M.; Bejger, G.; Yang, B.; Gelin, S.; Alem, N.; Dabo, I.; Rost, C. M.; Sinnott, S. B.; Crespi, V.; Gopalan, V.; Engel-Herbert, R.; Heron, J. T.; Maria, J.-P. Chemically-Disordered Transparent Conductive Perovskites With High Crystalline Fidelity. *Advanced Science*, e09868. https://doi.org/10.1002/advs.202509868.

(14) Spurling, R. J.; Almishal, S. S. I.; Casamento, J.; Hayden, J.; Spangler, R.; Marakovits, M.; Hossain, A.; Lanagan, M.; Maria, J.-P. Dielectric Properties of Disordered A6B2O17 (A = Zr; B = Nb, Ta) Phases. *Journal of the American Ceramic Society* **2024**, *107* (10), 6868–6875. https://doi.org/10.1111/jace.19966.

(15) Mazza, A. R.; Skoropata, E.; Sharma, Y.; Lapano, J.; Heitmann, T. W.; Musico, B. L.; Keppens, V.; Gai, Z.; Freeland, J. W.; Charlton, T. R. Designing Magnetism in High Entropy Oxides. *Advanced Science* **2022**, *9* (10), 2200391.

(16) Yoo, S.; Chae, S.; Chiang, T.; Webb, M.; Ma, T.; Paik, H.; Park, Y.; Williams, L.; Nomoto, K.; Xing, H. G.; Trolier-McKinstry, S.; Kioupakis, E.; Heron, J. T.; Lu, W. D. Efficient Data Processing Using Tunable Entropy-Stabilized Oxide Memristors. *Nat Electron* **2024**, *7* (6), 466–474. https://doi.org/10.1038/s41928-024-01169-1.
